# Water supplementation after dehydration improves judgment and decision-making performance

**DOI:** 10.1007/s00426-018-1136-y

**Published:** 2019-01-21

**Authors:** Olivia C. Patsalos, Volker Thoma

**Affiliations:** 1 Section of Eating Disorders, Department of Psychological Medicine, King’s College London, London, SE5 8AZ UK; 2 University of East London, Stratford Campus, Water Lane, London, E15 4LZ UK

## Abstract

Previous research has shown that dehydration and water supplementation affect mood and cognitive performance in both adults and children on a variety of tasks that assess memory, attention, executive function, and speeded responses. Given the varied effects of water on cognition, this study explored potential effects of water supplementation, hydration status, and thirst on thinking and decision-making tasks. 29 adult participants undertook a battery of cognitive tests on two separate occasions after having fasted from the previous night. On one occasion, they were offered 500 ml of water to drink prior to testing. Measures of urine osmolality confirmed the group-level effectiveness of the dehydration manipulation. Water supplementation was found to improve performance on tasks measuring cognitive reflection in judgement and decision-making. This increase in performance was associated with differences in tasks implicated in inhibition processes. Drinking water after a 12-h dehydration period increased performance in judgement and decision-making tasks, and this was not explained by differences in subjective thirst or attentiveness.

## Introduction

Several studies have found that dehydration affects not only mood but cognitive performance in both adults and children (see Benton, 2011 for review). For example, Sharma et al. (1986), who dehydrated their participants to 1%, 2% and 3% loss of body weight, found that performance on a memory test and psychomotor stylus test worsened with increasing levels of dehydration. Similarly, exercise-induced dehydration of 2% loss of body weight was found to negatively affect performance on memory tasks, mathematical calculation tasks, and executive function tasks (Gopinathan, Pichan, & Sharma, 1988). More recently, Benton, Jenkins, Watkins, and Young (2016) reported improved memory and focussed attention in individuals dehydrated to 1% body loss. Furthermore, studies investigating the effects of dehydration in children have found that their performance on memory tasks is negatively affected (Bar-David, Urkin, & Kozminsky, 2005; Fadda et al., 2012).

In addition to dehydration studies, the effects of water consumption on cognitive performance have been examined separately. Edmonds, Crombie, Ballieux, Gardner, and Dawkins (2013) found that water consumption resulted in improved performance on a visual attention task at both 20 min and 40 min after water supplementation when compared to baseline scores. In similar studies conducted in children, water supplementation was found to improve performance on memory and attention tasks (Edmonds & Jeffes, 2009; Edmonds & Burford, 2009) and on a letter cancellation task, which assesses visual attention (Booth, Taylor, & Edmonds, 2012).

Some researches have been conducted in which the effects of thirst have also been evaluated. In one study, it was found that participants self-reporting as being very thirsty showed a dose-dependent improvement on a sustained attention task when drinking water (Rogers et al., 2001). More surprisingly, though, participants who reported a low thirst level showed a dose-related impairment in performance on the same task. The role of thirst on cognitive performance was further evidenced in a study by Edmonds et al. (2013), in which it was reported that thirst facilitated performance on tasks requiring controlled processing (set shifting). This study also reported that thirsty participants performed significantly better on a simple reaction time task after drinking water and that this was moderated by participants’ subjective feelings of thirst.

In light of these findings, it is surprising that to the best of our knowledge, no studies have been investigating the impact of dehydration and water supplementation on judgement and decision-making, crucial tasks in everyday life. This is even more surprising, as there are clear links between executive functions and judgment and decision-making performance (Cokely & Kelley, 2009). For example, Toplak, West, and Stanovich (2011) found that the cognitive reflection test (CRT) correlates positively with working memory, inhibition, and set-shifting performance. Hence, in the present investigation, we have employed tasks that typically tap judgement and decision-making processes, aiming to capture any potential effects of water supplementation and thirst on thinking and reasoning abilities when participants are in a dehydrated state.

People are faced daily with judgements and decisions which potentially put great demands on our cognitive processes. Consequently, people often rely on heuristics (mental shortcuts) that simplify the task at hand—even in cases in which the task is a relatively simple one to solve (Frederick, 2005). A sizeable body of research suggests that heuristics can lead to systematic deviations from logic, resulting in predictable biases and inconsistencies (Kahneman, 2003). When faced with certain probabilistic judgment problems, people tend to use heuristics instead of reflective thinking that resembles an ‘algorithmic’ approach—the considered use of rule-based processes that should lead to normatively correct outcomes (though see Gigerenzer & Goldstein, 1996 for a different view of heuristics). This led to a distinction between automatic (heuristic) and reflective (analytic) thinking, described as ‘System 1’ (or ‘Type 1’ processing, Evans, 2008) and ‘System 2’ (or ‘Type 2’ processing, Evans, 2003; Sloman, 1996).

To be able to measure what happens when Type 1 processing is in conflict with the more reflective Type 2 processing, Frederick (2005) developed the CRT. This brief test consists of short maths puzzles, which can appear at first glance as having a very obvious answer. However, the respondent’s initial intuitive response is incorrect, and they can only arrive at the correct response if they suppress their initial heuristic answer and engage in more reflective thinking. The CRT has been linked to performance on numerous judgment and decision-making tasks, such as risk taking, temporal discounting, and use of heuristic thinking (Frederick, 2005; Toplak, Stanovich, & West, 2011).

Hydration has been previously linked to activation in the anterior cingulate cortex (ACC), and the latter in turn has been proposed to be an important component of frontal attentional control systems (Braver, Barch, Gray, Molfese, & Snyder, 2001; Bush et al., 1998; Pardo, Pardo, Janer, & Raichle, 1990). We therefore employed a number of executive function tasks to test possible links between hydration and executive functions such as inhibition, set shifting and updating processes (Miyake et al., 2000). Research has shown that the performance on the CRT and heuristics thinking vignettes may also depend on executive functions, such as inhibition capability (De Neys & Glumicic, 2008). Specifically, if overcoming automatic thinking patterns (such as the ‘implied’ but incorrect answers in the heuristic vignettes and the CRT) is due to inhibition (of automatic—‘intuitive’ responses), then we predicted that performance on an alternative choice reaction time task (ChoiceRT) would affect the relationship between water supplementation and cognitive reflection scores. This was measured in the current study with the ChoiceRT, which measures Stroop-like inhibition performance (we used the Cambridge Neuropsychological Test Automated Battery (CANTAB) software package (Sahakian & Owen, 1992) to assess cognitive performance, see Methods). In addition to inhibition processes, judgement and decision-making performance may also rely on the ability to symbolically manipulate mental representations (see Buckner & Carroll, 2007) and deal with calculations (Toplak et al., 2011). Hence, we also employ a set-shifting task, the CANTAB Intra-Extra Dimensional Set Shift (IED), which measures cognitive flexibility and the ability to keep mental concepts or representations separate. Finally, solving judgement vignettes may also require sustained attention (see Booth et al., 2012) and regular updating of working memory content (Miyake et al., 2000), as measured with the CANTAB Rapid Visual Processing task (RVP), in which participants have to monitor a sequence of numbers and respond when observing a target sequence of three consecutive numbers.

The aim of this study was to investigate the effects of hydration status, drinking water, and thirst on a range of cognitive processes. Based on the findings of previous studies (Benton & Burgess, 2009; Edmonds & Jeffes, 2009, Booth et al., 2012, Edmonds et al. 2013, Rogers et al., 2001, Benton et al., 2016), we expected performance for sustained attention and executive function tests to be positively affected by water consumption (Hypothesis 1). It was predicted that since water consumption has positive effects on typical cognitive processing tests, this should extend to judgment and decision-making tasks (Hypothesis 2) and that attention and executive performance scores (here: set shifting) correlate with judgement and decision-making tasks (Hypothesis 3). If so, judgement and decision-making scores may be differentially associated—depending on water supplementation—with scores of tasks measuring general levels of attention (Hypothesis 4a), mental manipulation of information (Hypothesis 4b) or inhibition performance (Hypothesis 4c). The last two predictions are mainly based on the dual process theories predicting monitoring of type 1 processing by a reflective system that either inhibits automatic thinking processes (Frederick, 2005; Kahneman, 2011), increases thinking performance in terms of mental manipulation abilities, or both (Toplak et al., 2011).

Importantly, we controlled for whether these effects were moderated by thirst or physiological dehydration status. Urinary osmolality analysis was used to assess hydration status to formally examine a potential association between hydration status, thirst, drinking water and cognitive performance.

## Methods

### Participants

31 participants (14 males) were recruited for this study through advertisements placed at the University of East London (UEL) and on psychology websites, via emails to UEL students, and through friends. A pre-participation health questionnaire was sent to interested individuals, to exclude persons for whom overnight fasting might have been a potential health risk (e.g., pregnant women and people suffering from diabetes or a heart condition). The ages of participants ranged from 21 to 46 years (mean 31 years; SD 7.24).

### Tasks and questionnaires

Task were presented to participants in the following order. Parallel forms of all tasks were administered counterbalanced across water condition and task version.

#### International positive and negative affect schedule short form (I-PANAS-SF)

The I-PANAS-SF scale is a shorter version of the original PANAS consisting of 10 items instead of 20 (Thompson, 2007) used to measure general affect. Half of the emotion words presented reflect negative affect states (ashamed, afraid, hostile, nervous, upset) and the other half reflect positive affect states (active, alert, attentive, determined, inspired). Participants rated their positive and negative affect on a 5-point scale that ranged from “very slightly or not at all” (1) to “extremely” (5).

#### Thirst scale

Participants were asked to indicate their level of thirst by marking an X on a continuous horizontal line (17.8 cm) with anchors indicating “not at all” to “very thirsty” (Edmonds et al., 2013). This was converted to a percentage where a higher percentage indicated a higher level of thirst.

#### Cambridge neuropsychological test automated battery (CANTAB)

The CANTAB eclipse software (Sahakian & Owen, 1992) contains an array of tests used to assess cognitive performance. We administered six tests from this platform: the motor screening test (MOT), the simple reaction time (SRT), the choice reaction time (ChoiceRT), the big/little circle (BLC), the intra-extra dimensional set shifting (IED), and the rapid visual information processing (RVP). The IED, RVP and ChoiceRT assess executive functions including visual attention, which is the focus of this report.

ChoiceRT is a 2-choice reaction time test with stimulus and response uncertainty introduced by having two possible stimuli and two possible responses. Participants were instructed to press the left-hand button if the stimulus (an arrow) was displayed on the left-hand side of the screen, and the right-hand button if the stimulus was displayed on the right-hand side of the screen. A practice stage (24 trials) was followed by two assessment stages (50 trials each). The dependent variable was reaction time.

IED is a test of rule acquisition and reversal. Two patterns are displayed on the screen, first simple (colour-filled shapes) and then compound (white lines overlying colour-filled shapes). The participant must learn which of the two stimuli was correct by trial and error learning. When six consecutive correct responses were recorded, the contingencies were reversed and this pattern of stimulus addition and reversal continued for nine blocks. If the participant failed to reach six consecutive responses after 50 trials, the test was terminated. The dependent variable for this task was the total errors committed.

RVP is a sensitive measure of general performance and in particular of visual sustained attention. Numbers appear one at a time in a box in the centre of the screen at the rate of 100 digits per minute. Participants were instructed to press the button on the press pad whenever they spotted a target sequence of three consecutive numbers. A practice stage (lasting 2 min) in which participants were prompted as to when a sequence had begun and when to press the button was followed by a test stage (lasting 4 min) in which no cues were displayed and the participant had to spot three different sequences on their own. Target sequences occurred at the rate of 16 every 2 min. The measured dependent variable was total error rate.

#### Measuring cognitive reflection performance

To assess judgement and decision-making performance, we employed tasks that are typically used to assess the use of heuristic (automatic) processing that can be overcome by reflective (controlled and analytic) thinking (following largely Toplak et al., 2011). This consisted of nine vignettes or puzzles in total per session. Six of these were heuristics-and-biases vignettes from widely cited publications that reflect important aspects of rational thought such as probabilistic reasoning, hypothetical thought, theory justification, scientific reasoning, and the tendency to think statistically. Each answer to a heuristic vignette task was scored as correct or incorrect (1 or 0 score), resulting in a total maximum score of 6 (per session). The battery was comprised of the following:

1. Causal base rate (Fong, Krantz, & Nisbett, 1986).
2. Sample size (Tversky & Kahneman, 1974).
3. Gambler’s fallacy (Toplak et al., 2011).
4. Conjunction fallacy (Tversky & Kahneman, 1983).
5. Bayesian reasoning (Doherty & Mynatt, 1990).
6. Sunk cost (Arkes & Blumer, 1985).

Example of sample size:

A certain town is served by two hospitals. In the larger hospital about 45 babies are born each day, and in the smaller hospital about 15 babies are born each day. As you know, about 50% of all babies are boys. However, the exact percentage varies from day to day. Sometimes it may be higher than 50%, sometimes lower. For a period of 1 year, each hospital recorded the days on which more than 60% of the babies born were boys. Which hospital do you think recorded more such days?

- The larger hospital.
- The smaller hospital.
- About the same (that is, within 5% of each other).

In addition to the vignettes inducing heuristic thinking, the Cognitive Reflection Test (CRT; Frederick, 2005) was used. The CRT is designed to measure participants’ tendency to override an intuitive first response and to engage in reflective thinking to arrive at the correct answer (similar to the mechanism proposed to work in solving heuristic vignettes, Kahneman, 2011). The dependent variable was the total number of correct responses (maximum of 3 per session). The original CRT comprised of only three questions. We used the extended version by Toplak et al. (2014) resulting in different three questions in each of the two sessions. The answers to the six heuristic vignettes and the three CRT puzzles formed the cognitive reflection score (a maximum of nine correct answers per session). An example of the CRT is the following: in a lake, there is a patch of lily pads. Every day, the patch doubles in size. If it takes 48 days for the patch to cover the entire lake, how long would it take for the patch to cover half of the lake? (Intuitive answer: 24; correct answer: 47).

### Measurement of hydration status

A Vitech Advanced Multi Sample Micro freezing point osmometre from Advanced Instruments Inc. was used to determine urine osmolality (mOsm/kg) to assess participants’ hydration status. A higher value indicates a greater degree of dehydration. According to the US National Institutes of Health, a concentration of 500–800 mOsm/kg is considered normal, whereas a 12–14 h fluid restriction should yield a value in excess of 850 mOsm/kg (Chernecky & Berger, 2012). A higher value indicates a greater degree of dehydration.

### Procedure

A pre-participation health questionnaire was sent to interested individuals, to exclude persons for whom overnight fasting might have been a potential health risk (e.g., pregnant women and people suffering from diabetes or a heart condition). They were also provided with an empty sample container in which they supplied their waking urine sample, which they also brought with them to each of their sessions.

Participants visited UEL’s Psychology Research Suite on two occasions, 1 week apart, after having fasted (no food or drink) from 9 p.m. the night before. Participants were asked to collect a urine sample upon waking (in sterile sample pots already provided), which they brought with them. Testing took place in the mornings (8 a.m.–11 a.m.). To standardise the water content of breakfast, before each testing session, participants received a choice of cereal bar (113 kcal or 119 kcal). On one occasion (counterbalanced across participants), they were also given a 500 ml bottle of water (at room temperature). Participants were explicitly and clearly instructed to drink as much as they wanted before beginning the tasks. There was no time pressure, but all participants stopped drinking after 2 min. They were not allowed to continue drinking during testing.

Participants then completed the tasks in the order they have been described above. At the end of testing, they were asked to provide another urine sample. The second session followed the same procedure and at the end of the second session they were debriefed and compensated for their time and participation. Tasks in both sessions were completed in approximately 1 h.

The order of water supplementation and tasks administered was counterbalanced so that 15 participants had water in their first session and 14 in their second session, and 15 had version A of decision-making tasks in their first session and 14 had version B of decision-making tasks in their second session.

### Data analysis

The main aim of this study was to investigate the effect of water supplementation on cognitive performance. To test hypothesis 1 and 2, the data was subjected to a series of mixed analyses of variance (ANOVA) in which water supplementation (water/no water given) was a within-participants factor, and order (water first/no water first), thirst (thirsty/not thirsty), and urine osmolality (high/low) were between-participants factors. The same analyses were also performed for the combined cognitive reflection scores.

For thirst and hydration, median splits were performed grouping participants as either thirsty/not thirsty and hydrated/not hydrated based on the respective medians of 63% and 827.5 mOsm/kg on the ‘no water day’. The post-test osmolality data was used in the present analyses. The pre-test data was used to confirm fasting (see “Results”).

To investigate hypothesis 3 and 4, correlation analyses were also performed in an attempt to tease apart a possible relationship between performance on the judgment and decision-making tasks and performance on the ChoiceRT, IED, and RVP.

## Results

Data from two participants could not be analysed because they did not return for the second session. The final sample size was 29 participants (16 females).

### Water consumption and hydration status effects on thirst and mood scales

**Fig. 1:**
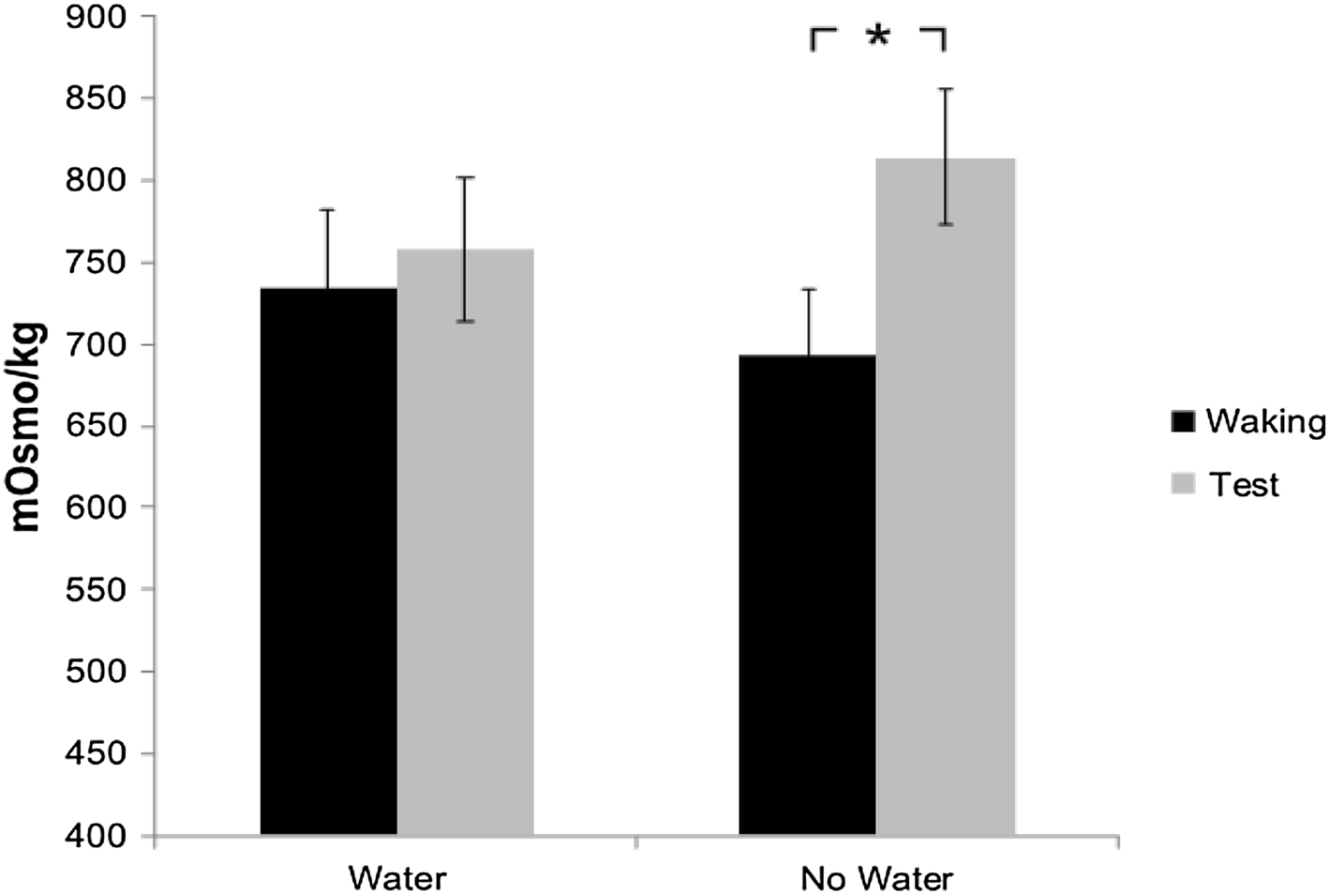
Mean osmolality readings for participants on different sessions and time points during testing days (before—“waking”—and after tests). Error bars are standard errors of the mean. Significant differences (using “asterisk” to denote *p* > .05) between conditions are indicated

In the water condition, participants drank a mean of 303.44 ml (SD 158.21; range 50–500 ml). To test whether people were indeed dehydrated in the no water condition after test as well as on both mornings, we ran a 2 (water vs no water) by 2 (waking vs end of test) ANOVA on osmolality readings. There was no effect of day, *F*_(1,28)_ < 1, but a main effect of test time, (*F*_(1,28)_ = 5.96, *p* = .021, *η*_*p*_^2^ = 0.176), as well as an interaction, (*F*_(1,28)_ = 6.231, *p* = 0.019, *η*_*p*_^2^ = 0.182). Whereas there was no difference between hydration readings in the water condition before (*M* = 735, SD = 252) and after (*M* = 758, SD = 235) testing, there was a difference in the no water day, with readings lower before (*M* = 693, SD = 218) than after (*M* = 813, SD = 217) testing (see Fig. 1). This suggests that on a group level, participants were reasonably dehydrated (osmolality readings of ca 700–800 mOsmo/kg), but also that in the no water day, the dehydration became significantly worse during the morning compared to the water day (Edmonds et al., 2013). Thus, water supplementation on the water day prevented further dehydration, which seemed to happen on the no water day as testing went on through the morning. Thirst ratings also confirmed that participants arrived thirsty: participants rated themselves as having greater subjective thirst on the occasion that they were not offered water (*F*_(1,27)_ = 46.112, *p* < 0.001).

**Fig. 2:**
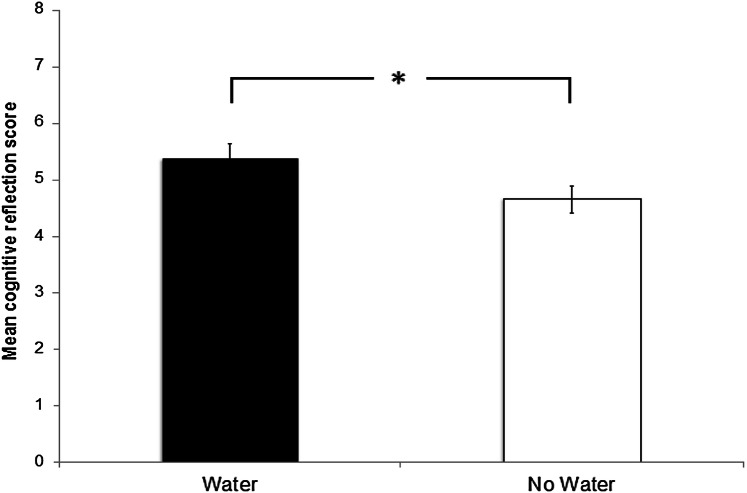
Mean scores for the combined judgment and decision-making tasks comparing performance on the day participants received water with the day they did not. Water consumption has a significant effect on scores, with participants scoring better on the day they did receive water

The responses to the I-PANAS-SF mood scale were mostly unaffected by water supplementation, thirst, order and osmolality. There were two exceptions to this statement: there was a water supplementation x order interaction for “attentive” and an osmolality effect on “inspired”. Participants who received water in their first session reported being more “attentive” on that occasion compared to their second session in which they did not have any water (*F*_(1,27)_ = 16.00, *p* < 0.001). In the case of urine osmolality, dehydrated participants (as evidenced by higher urine osmolality) rated themselves as significantly less “inspired” (*F*_(1,27)_ = 4.276, *p* = 0.048). There was no effect of thirst (high vs low scorers) on any of the items presented in the I-PANAS-SF mood scale.

### Water consumption effects on executive functions

Mean scores on CANTAB tests were screened for normal distribution and outliers, using the interquartile range rule of g = 3 (Hoaglin et al, 1986). Only one RVP errors data point was substituted with RVP misses in one condition for one participant who had a very high RVP false alarm rate in one condition. For all other participants, the RVP total errors were calculated as the sum of the number of false alarms and number of misses.

Performance on each of the CANTAB tasks was analysed using mixed-design ANOVAs, one separately for effects of order, thirst, and osmolality. The within-participants factor in each ANOVA was water supplementation (water vs no water). The between factors in the respective ANOVAs were order (water first session or water second session), hydration [osmolality: high > 827.5 mOsm/kg or low < 827.5 mOsm/kg; i.e., dehydrated (15) or hydrated (14)], and finally thirst (high or low after median split; 14 participants classified as thirsty and 15 as not thirsty). There were no significant effects or trends for the factor order or water (see Tables 1, 2, 3), bar two exceptions. There were trends for ChoiceRTs to be generally faster in the water conditions (*p* values between 0.066 and 0.073; Tables 1, 2, 3), and there was a significant interaction for water and order in the RVP tasks (Table 1), with more errors in the water condition (M = 17.80, SD = 4.72) compared to the no water condition (M = 21.13, SD = 5.55) when participants received water in their first session, *p* = 0.007, but vice versa when they received it second, *p* = 0.057 (water: M = 22.07, SD = 3.15; no water: M = 20.50, SD = 3.65). Therefore, hypothesis 1 could not be retained.

**Table 1:** CANTAB test means, SDs and F ratios by water condition (water/no water) and order (water first/no water first)

Task	Water first				No water first				Results from the omnibus statistical analysis; those with p < .05 in bold
	Water		No water		Water		No water		
	*M*	SD	*M*	SD	*M*	SD	*M*	SD	
ChoiceRT	317.81	51.63	336.33	74.21	288.73	31.55	292.27	35.05	Water *F*_(1,27)_ = 3.476, *p* = 0.073
									Water × order *F*_(1,27)_ = 1.600, *p* = 0.217
IED total errors	20.07	10.53	18.33	11.76	13.79	9.31	15.43	9.99	Water *F*_(1,27)_ = 0.002, *p* = 0.966
									Water × order *F*_(1,27)_ = 2.615, *p* = 0.117
RVP errors	10.53	5.64	7.60	6.34	5.50	3.43	7.42	4.21	Water *F*_(1,26)_ = 0.335, *p* = .568
									**Water** × **order*F***_**(1,26)**_ = **14.259**, ***p*** = **0.001**

**Table 2:** CANTAB test means, SDs and *F* ratios by water condition (water/no water) and post-testing urine osmolality (low/high) as measured on the day participants did not receive any water

Task	Low osmolality				High osmolality				Results from the omnibus statistical analysis; those with *p* < .05 in bold
	Water		No water		Water		No water		
	*M*	SD	*M*	SD	*M*	SD	*M*	SD	
ChoiceRT	304.26	46.83	319.05	75.54	303.31	44.61	311.34	48.19	Water *F*_(1,27)_ = 3.548, *p* = 0.070
									Water × osmo *F*_(1,24)_ = 0.312, *p* = 0.581
IED total errors	17.53	11.04	18.67	11.32	16.50	9.80	15.07	10.41	Water *F*_(1,27)_ = 0.019, *p* = 0.891
									Water × osmo *F*_(1,24)_ = 1.446, *p* = 0.240
RVP errors	8.57	5.71	7.64	5.51	7.71	5.21	7.50	5.53	Water *F*_(1,26)_ = 0.552, *p* = 0.464
									Water x Osmo *F*_(1,26)_ = 0.169, *p* = 0.684

**Table 3:** CANTAB test means, SDs and *F* ratios by water condition (water/no water) and thirst (low/high) as measured on the day participants did not receive any water

Task	Low thirst				High thirst				Results from the omnibus statistical analysis; those with *p* < .05 in bold
	Water		No water		Water		No water		
	*M*	SD	*M*	SD	*M*	SD	*M*	SD	
ChoiceRT	303.96	41.45	309.75	38.81	303.57	49.87	320.75	80.94	Water *F*_(1,27)_ = 3.676, *p* = 0.066
									Water × thirst *F*_(1,24)_ = 0.903, *p* = 0.350
IED total errors	16.29	10.61	15.43	10.17	17.73	10.31	18.33	11.62	Water *F*_(1,27)_ = 0.011, *p* = 0.916
									Water × thirst *F*_(1,27)_ = 0.124, *p* = 0.728
RVP errors	8.14	5.75	7.64	4.73	8.14	5.20	7.50	6.21	Water *F*_(1,27)_ = 0.536, *p* = 0.470
									Water × thirst *F*_(1,27)_ = 0.024, *p* = 0.879

Regarding the main effects of between-subjects variables, there was a marginal effect of order on the ChoiceRT, *F*_(1,27)_ = 4.034, *p* = .055, with higher RTs in the first session (M = 327, SD = 12) than in the second (M = 290, SD = 13). Otherwise, there were no effects, all *Fs* < 1, except for IED errors and order, *F*_(1,27)_ = 1.503, *p* = .23, and order effects on RVP error rates, *F*_(1,27)_ = 2.161, *p* = .153. Controlling for the amount of water each participant drank (using ANCOVAs) did not change the pattern of effects, all *F*s_*(1,28)*_ < 1, except for a similar trend as above for ChoiceRT *F*_(1,28)_ = 3.53, *p* = .71. Additional ANCOVAs using thirst and osmolality as co-variates (rather than median split as a between-group factor), again found similar effects for the independent variables on CANTAB scores, all *F*s < 1 (except the trend of water for ChoiceRT, with *p* values between 0.065 and 0.071.

### Water consumption effects on judgment and decision-making performance

Three mixed-design ANOVAs were performed with total correct score for the combined judgement and decision-making tasks (six heuristic vignettes and three CRT vignettes in each session, see Methods) as the dependent variable, analogous to the ANOVAs for the executive function tests above. The within-participant factor in each ANOVA was water supplementation (water vs no water). The between factors in the respective ANOVAs were order (water first session or water second session), hydration (osmolality: high or low; i.e., dehydrated or hydrated), and finally thirst (high or low after median split). In all three ANOVAs, there was a main effect of water supplementation (Table 4), for the ANOVA on water and order (*F*_(1,27)_ = 7.37, *p* = 0.011, *η*_*p*_^2^ = 0.215), water and hydration (*F*_(1,27)_ = 7.44, *p* = 0.013, *η*_*p*_^2^ = 0.209), and water and thirst (*F*_(1,27)_ = 7.69 *p* = 0.012, *η*_*p*_^2^ = 0.212). Participants scored overall higher on the judgment and decision-making tasks in conditions in which they received water compared to the no water day (Fig. 2). There were no simple main effects from factors order, *F*_(1,27)_ 2.730, *p* = .110, hydration *F*_(1,27)_ < 1, or thirst, *F*_(1,27)_ = 2.33, *p* = .138. There were also no interaction effects involving order, all *F*s < 1. Water supplementation therefore had a positive effect on scores across the battery of judgment and decision-making tasks, relatively independent of levels of thirst and hydration (on the no water day), or order. The ANCOVAs using thirst and osmolality (mean-centred) as co-variates instead of median splitting found the same patterns effects on cognitive reflection scores, with no main or interaction effect of the co-variates (all *Fs* < 1).

**Table 4:** Cognitive reflection score means, SDs and *F* ratios by water condition (water/no water) and post-testing urine osmolality (low/high) as measured on the day participants did not receive any water

	Water	No water			Results from the statistical analysis
	*M*	SD	*M*	SD	
Water first	4.80	2.18	4.27	1.62	Water *F*_(1,27)_ = 7.374, *p* = 0.011, *η*_*p*_^2^ = 0.215
Water second	6.00	1.47	5.07	1.77	Water × order *F*_(1,27)_ = 0.539, *p* = 0.469
Osmo low	5.47	2.44	4.53	1.92	Water *F*_(1,27)_ = 7.439, *p* = 0.013, *η*_*p*_^2^ = 0.209
Osmo high	5.29	1.27	4.79	1.53	Water × osmo *F*_(1,27)_ = 0.651, *p* = 0.427
Thirst low	4.87	2.13	4.27	1.53	Water *F*_(1,27)_ = 7.688, *p* = 0.012, *η*_*p*_^2^ = 0.212
Thirst high	5.93	1.59	5.07	1.86	Water × thirst, *F*_(1,27)_ = 0.226, *p* = 0.639

This result confirmed hypothesis 2, that water supplementation increased cognitive reflection scores, and this result was not qualified by any interaction.

### Correlation analysis

To investigate the possible relationship between hydration variables, judgment scores and executive functions for different water supplementation conditions, we performed correlation analyses. Table 5 shows differing degrees of associations depending on whether the data used was taken from the day participants received water or not.

**Table 5:** Correlations between cognitive reflection performance scores and water consumption, urine osmolality, thirst, CANTAB tasks and for both days (participants received/did not receive water) (*N* = 29)

Measured variable	Water	No water
Water consumed (ml)	0.208	–
Urine osmolality (post)	0.132	0.107
Thirst	−0.168	0.169
ChoiceRT (RT)	−0.473**	−0.579***
IED (errors)	−0.533**	−0.578**
RVP (errors)	−0.451*	−0.148

*p ≤ 0.05. **p < .01. ***p < .001

There were significant correlations between cognitive reflection scores and ChoiceRT (water: *r* = − 0.473, *p* = 0.010; no water: *r* = − 0.579, *p* = 0.001) and IED errors (water: *r* = − 0.533, *p* = 0.003; no water: *r* = − 0.578, *p* = 0.001) on both days, water and no water, respectively. There was also a significant correlation between CRT scores and RVP errors on the water day only (*r* = − 0.451, *p* = 0.014). All correlations were in the predicted direction with better performance (lower errors or shorter RTs) in executive function tasks being associated with higher cognitive reflection performance. Hypothesis 3 was therefore confirmed—performance on executive function tasks (though only in the water condition for RVP) was associated with higher performance in the judgment and decision-making tasks.

Finally, a linear regression analysis was performed using difference scores (water–no water). The difference in cognitive reflection scores between the no water and the water condition served as the dependent variable (criterion) and the difference (between water and no water day) in ChoiceRT and IED errors as independent variables. The regression model tested whether differences (between sessions) in the executive function tasks were associated with differences in the cognitive reflection scores. Recall that the hypothesis was based on the premise by dual process theories that increased inhibition processes are related to increased performance in CRT-like puzzles and heuristic vignettes. Some approaches in the dual systems framework (e.g., Evans & Stanovich, 2013) further implicate mental simulation performance, the ability to maintain and symbolically manipulate separate mental representations of a problem.

ChoiceRT latency difference scores were log-transformed to reduce potential issues of positive skew and normality of residuals. Results of the multiple linear regression indicated that there was a combined significant effect of differences in ChoiceRT and IED (errors) explaining differences in cognitive reflection scores, (*F*_(2,26)_ = 3.765, *p* = 0.037, *R*^*2*^ = 0.224). ChoiceRT difference (*t* = − 2.244, *p* = 0.034) was a significant predictor in the model, but not IED error difference (*t* = − 1.543, *p* = 0.135) (Table 6). Adding RVP errors (difference scores) as a predictor variable again showed a relationship for cognitive reflection scores with ChoiceRT (*t* = − 2.343, *p* = 0.027) but not RVP errors (*t* = − 1.005, *p* = 0.324), with the overall model marginally significant, (*F*_(2,25)_ = 4.899, *p* = 0.058, *R*^2^ = 0.254). Thus, hypothesis 4c was retained: cognitive reflection scores for sessions in which water was given were differentially influenced by ChoiceRT scores compared to sessions in which water was not given—the higher the differences in ChoiceRT latencies (and therefore the worse the inhibition performance between no water and water condition), the lower the improvement of cognitive reflection scores from no water to water condition.

**Table 6:** Summary of regression analysis for variables predicting cognitive reflection score differences (*N* = 29) between sessions

Source	*B*	SE *B*	*β*	*t*	*p*		LBCI 95%	UBCI 95%
ChoiceRT—diff	−4.357	1.942	−0.388	−2.244		0.034*	−8.349	−0.366
IED (errors)—diff	−0.066	0.043	−0.267	−1.543		0.135	−0.155	0.022

Hypothesis 4a and 4b were therefore not retained—differences in tasks measuring attention performance (RVP) or mental simulation (IED) between the water supplementation conditions were not associated with the difference in cognitive reflection scores.

## Discussion

The current study is to our knowledge the first to report increased cognitive reflection performance (and, by extension, increased judgment and decision-making performance, Frederick, 2005; Toplak et al., 2011) after water consumption. When thirst, hydration status, and mood state were controlled for, water supplementation increased performance on an overall composite score from widely used judgment and decision tasks (judgment vignettes eliciting heuristic thinking, simple maths puzzles requiring cognitive reflection). These scores were related to inhibition processing speed and executive functions (ChoiceRT and IED), but not attentional performance (RVP) or feelings of general attentiveness. The experimentally induced differences in judgement and decision performances between water days and no water days were associated with differences in Stroop-like task performances (and Simon task) generally associated with inhibition processes. Before we turn to these effects in detail, we discuss the physiological factors that could have influenced this result.

In general, there were no effects of water, thirst or hydration status (except for PANAS ‘attentive’ scores, but those were not associated with cognitive reflection performance) on the measures of mood used in this study. Some studies have previously reported links between dehydration and mood ratings (Shirreffs et al., 2004), and water supplementation and mood ratings (Edmonds et al., 2013), while others report that water supplementation does not affect mood (Edmonds et al., 2013). Furthermore, it may be that whether mood affects dehydration may depend on the manner in which dehydration is achieved: Shirreffs et al. (2004) induced dehydration by fluid restriction, whereas Edmonds et al. (2013) reported effects on water supplementation. At any rate, the main finding here is that mood (and hence expectation effects) does not explain the findings for the effect of water on cognitive reflection tasks.

Previous studies have revealed that both water supplementation and thirst impact on cognitive performance (Edmonds et al., 2013). However, we have failed to replicate the significant findings pertaining to participants’ subjective ratings of thirst as a moderator of the effects of water supplementation on most the measures assessed (including mood ratings). This could be an idiosyncrasy of the particular sample population or it could indicate individual differences in feelings of subjective thirst. For example, several participants (*N* = 7) in this study spontaneously expressed that they were seldom thirsty, so it was perhaps not surprising that even on the occasion when they were not given any water, they indicated a relatively low level of thirst. Nonetheless, as elucidated by the relevant statistical analysis on a group level, participants did report experiencing greater levels of subjective thirst on the occasion they did not receive any water.

Our main result is the significant effect of water supplementation on performance on the judgement and decision-making tasks (heuristics and biases, cognitive reflection test)—participants performed better on the occasion on which they received water. This finding cannot be easily dismissed as a result of demand characteristics (i.e., simply being given a drink increasing motivation, or expectation of doing better), because we did not find an influence of water supplementation on mood effects (see also Edmonds et al., 2013 who show that expecting water supplementation does not explain increased performance in attention tasks) nor on other cognitive tasks (IED, RVP). Therefore, we interpret the effects on cognitive reflection scores as substantially driven by water supplementation.

The tasks used here were aimed at assessing ‘slow’ processing (reflective thinking) vs ‘fast’ processing (heuristic thinking; Kahneman, 2003), with the particular aim to investigate potential processes that override decisions reached by Type 1 (De Neys & Glumicic, 2008). Inhibition performance has been shown to be influenced by water supplementation in previous research (Edmonds et al., 2013) and could thus modulate the effect of water supplementation on cognitive reflection performance. Indeed, performance on the Stroop-like ChoiceRT task correlated with judgment and decision performance score in both conditions, as was predicted (and replicated previous results, e.g., Toplak et al., 2011). In addition, regression analysis suggests a link between inhibition performance (as measured by the ChoiceRT) and the effect of water supplementation on decision performance: As ChoiceRT performance is affected by supplementation (although effect sizes are small), so is the performance on the heuristic vignettes and puzzles. Of course, we cannot directly infer causation, but it is noteworthy that most dual process theories of thinking and deciding (De Neys & Glumicic, 2008; Evans & Stanovich, 2013; Kahneman, 2011) predict that cognitive reflection performance relies on monitoring and consequently inhibiting the pre-potent responses related to heuristic thinking. It is the successful monitoring and inhibition that consequently decreases biased judgments.

Our finding that particular executive processes correlate with cognitive reflection tasks is also roughly in line with the psychobiological literature, especially the notion of a possible role of a behavioural inhibition in judgement performance. For example, fMRI studies found that dehydration directly affects the blood flow to the anterior cingulate cortex (Farrell et al., 2008), which is linked to inhibition (e.g., Stroop) performance. When comparing incongruent and control conditions, the majority of such studies report maximal differential activation occurring in the anterior cingulate cortex (Bench et al., 1993; Bush at al., 1998; Carter et al., 1995; Carter et al., 2000; Derbyshire, Vogt, & Jones, 1998; Pardo et al., 1990). Although this area shows greatest activation in the incongruent condition of the Stroop, the congruent condition (facilitation) has also been shown to increase activation as compared to a control condition (Bench at al., 1993; Carter et al., 1995). Thus, even though the choice response times may be deemed a somewhat indirect inhibition measure, both congruent and incongruent trials (and latency data) may indicate inhibition processes. Furthermore, previous work has shown that complex processing speed measures are substantially correlated with executive control measures but not with simpler speed measures (e.g., Cepeda et al., 2013). However, future research needs to further elucidate the exact mechanisms of inhibition and facilitation linked to water supplementation. Moreover, different tasks may tap into different inhibition processes. For example, Khng and Lee (2014) found performance on the Stroop tasks largely independent from performance on a Stop-signal task, indicating potentially different underlying inhibition processes. Although the ChoiceRT task employed here contains conditions that require Stroop-like inhibition processes, other tasks may help establish better models explaining the relationship between inhibition, executive functions, and cognitive reflection performance. In any case, though intriguing, the link between a task involving inhibition processes and cognitive reflection performance found here needs to be interpreted with caution until further replicated with other measures of inhibition.

In addition to inhibition, Toplak et al. (2011) found that the cognitive reflection task (CRT) also correlates with measures of cognitive ability (see also Stanovich & West, 2000). Similarly, Evans and Stanovich (2013) propose that the reflective system requires executive processes beyond inhibition to enable ‘cognitive decoupling’, that is the ability for mental simulation and abstract thinking. It is this ability that potentially allows the independent mental representation of information in math-like puzzles (CRT) and vignettes (heuristics) shown to participants. Indeed, our findings of strong correlations between IED (set shifting) and cognitive reflection tasks strongly indicates that some form of cognitive decoupling underlies Type 2-like processing. But here again, there was no indication that IED—as a proxy measure for mental simulation- modulates the relationship between water supplementation and cognitive reflection scores, unlike what we found with the ChoiceRT task.

Our findings are therefore the first that show a tentative link between water supplementation (after dehydration), inhibition performance, and judgement and decision-making processes. If future research confirms the effect of water supplementation (and a possible role of dehydration) on decision-making performance, the underlying cognitive–physiological mechanisms may be more complicated. Hydration has been linked to a range of inhibitory or excitatory effects, leading to cognitive improvements or impairments. For example, chronic dehydration in animals increases the release of the neurotransmitters gamma-aminobutyric acid (GABA) and glutamate, which have inhibitory and excitatory effects, respectively (Di & Tasker, 2004). Furthermore, dehydration has been shown to increase the release of the stress hormone cortisol (Francesconi et al., 1984), and elevated cortisol levels have been associated with impaired cognitive function (Greendale et al., 2000; Kirschbaum et al., 1996). In the present data, similar conflicting effects may therefore account for the difficulty in establishing stronger links between executive functions and cognitive reflection performance in different water supplementation (and hence hydration) conditions.

In conclusion, we find a clear effect of water supplementation (after dehydration) on decision-making performance when thirst is controlled for. The challenge for future studies will be to further clarify the relationship between physiological and cognitive mechanisms. Researchers will need to employ executive function tests that are sensitive to different types of inhibition mechanisms and other executive processes, as well as measuring effects stemming from physiological hydration and thirst.
